# Exome Sequencing Reveals a Putative Role for HLA-C*03:02 in Control of HIV-1 in African Pediatric Populations

**DOI:** 10.3389/fgene.2021.720213

**Published:** 2021-08-26

**Authors:** Samuel Kyobe, Savannah Mwesigwa, Grace P. Kisitu, John Farirai, Eric Katagirya, Angella N. Mirembe, Lesego Ketumile, Misaki Wayengera, Fred Ashaba Katabazi, Edgar Kigozi, Edward M. Wampande, Gaone Retshabile, Busisiwe C. Mlotshwa, Lesedi Williams, Koketso Morapedi, Ishmael Kasvosve, Jacqueline Kyosiimire-Lugemwa, Betty Nsangi, Masego Tsimako-Johnstone, Chester W. Brown, Moses Joloba, Gabriel Anabwani, Lukhele Bhekumusa, Sununguko W. Mpoloka, Graeme Mardon, Mogomotsi Matshaba, Adeodata Kekitiinwa, Neil A. Hanchard

**Affiliations:** ^1^Department of Medical Microbiology, College of Health Sciences, Makerere University, Kampala, Uganda; ^2^Department of Immunology and Molecular Biology, College of Health Sciences, Makerere University, Kampala, Uganda; ^3^Baylor College of Medicine Children’s Foundation, Kampala, Uganda; ^4^Botswana-Baylor Children’s Clinical Centre of Excellence, Gaborone, Botswana; ^5^School of Allied Health Professions, Faculty of Health Sciences, University of Botswana, Gaborone, Botswana; ^6^The Medical Research Council (MRC)/UVRI & LSHTM Uganda Research Unit, Entebbe, Uganda; ^7^Australian Medical Research, Hurstville, NSW, Australia; ^8^University of Tennessee Health Science Center, Memphis, TN, United States; ^9^Eswatini - Baylor College of Medicine Children’s Foundation, Mbabane, Eswatini; ^10^Department of Molecular and Human Genetics, Baylor College of Medicine, Houston, TX, United States; ^11^Department of Pathology and Immunology, Baylor College of Medicine, Houston, TX, United States; ^12^Pediatric Retrovirology, Department of Pediatrics, Baylor College of Medicine, Houston, TX, United States

**Keywords:** AIDS, childhood HIV, long-term non-progression, genetics, genomics

## Abstract

Human leucocyte antigen (HLA) class I molecules present endogenously processed antigens to T-cells and have been linked to differences in HIV-1 disease progression. HLA allelotypes show considerable geographical and inter-individual variation, as does the rate of progression of HIV-1 disease, with long-term non-progression (LTNP) of disease having most evidence of an underlying genetic contribution. However, most genetic analyses of LTNP have occurred in adults of European ancestry, limiting the potential transferability of observed associations to diverse populations who carry the burden of disease. This is particularly true of HIV-1 infected children. Here, using exome sequencing (ES) to infer HLA allelotypes, we determine associations with HIV-1 LTNP in two diverse African pediatric populations. We performed a case-control association study of 394 LTNPs and 420 rapid progressors retrospectively identified from electronic medical records of pediatric HIV-1 populations in Uganda and Botswana. We utilized high-depth ES to perform high-resolution HLA allelotyping and assessed evidence of association between HLA class I alleles and LTNP. Sixteen HLA alleles and haplotypes had significantly different frequencies between Uganda and Botswana, with allelic differences being more prominent in HLA-A compared to HLA-B and C allelotypes. Three HLA allelotypes showed association with LTNP, including a novel association in HLA-C (HLA-B^∗^57:03, aOR 3.21, *Pc* = 0.0259; B^∗^58:01, aOR 1.89, *Pc* = 0.033; C^∗^03:02, aOR 4.74, *Pc* = 0.033). Together, these alleles convey an estimated population attributable risk (PAR) of non-progression of 16.5%. We also observed novel haplotype associations with HLA-B^∗^57:03-C^∗^07:01 (aOR 5.40, *Pc* = 0.025) and HLA-B^∗^58:01-C^∗^03:02 (aOR 4.88, *Pc* = 0.011) with a PAR of 9.8%, as well as a previously unreported independent additive effect and heterozygote advantage of HLA-C^∗^03:02 with B^∗^58:01 (aOR 4.15, *Pc* = 0.005) that appears to limit disease progression, despite weak LD (*r*^2^ = 0.18) between these alleles. These associations remained irrespective of gender or country. In one of the largest studies of HIV in Africa, we find evidence of a protective effect of canonical HLA-B alleles and a novel HLA-C association that appears to augment existing HIV-1 control alleles in pediatric populations. Our findings outline the value of using multi-ethnic populations in genetic studies and offer a novel HIV-1 association of relevance to ongoing vaccine studies.

## Introduction

HIV-1 remains a significant public health concern in Africa, with an estimated 26 million people living with HIV-1 in the region (UNAIDS, 2019). Despite the introduction of effective antiretroviral therapy (ART) and the roll-out of prevention of mother-to-child transmission programs, more than 70% of all new childhood infections occur in Africa, numbering close to 220,000 new HIV-1 infections and accounting for more than 110,000 deaths in children annually on the continent (UNAIDS, 2019). However, 1–5% of children infected with HIV-1 do not progress to AIDS for more than 10 years even without ART (Warszawski et al., 2007) – so-called long-term non-progressors (LTNPs). We have documented a cohort of childhood LTNPs from Uganda and Botswana (Kyobe et al., under review) having one of the highest documented frequencies of LTNPs (8%) in both countries. In general, LTNPs possess a high degree of HIV-1 infection control with significant viral suppression and high CD4 + T cell count (>500 cells/mL) (Warszawski et al., 2007), and have lower levels of the immune activation markers such as HLA-DR and CD38 despite chronic infection (Hua et al., 2014); this pattern is frequently associated with the expression of broadly neutralizing antibodies (bNAbs) (González et al., 2018).

HLA class I molecules predominantly function to present endogenously processed antigens to cytotoxic CD8 + T lymphocytes (CTL) in a cell-mediated immune response (Frater et al., 2007), and play a significant role in the innate immune response through interactions with natural killer (NK) cell receptors via the killer cell immunoglobulin-like receptors (KIRs) (Boudreau and Hsu, 2018). The latter interaction puts class I molecules at the epicenter of HIV-1 control and makes them a focal point for developing and designing epitope-based vaccines (Kaseke et al., 2021). As a result, genetic variation at HLA class I has been frequently associated with LTNP status. Several studies, mainly conducted among adults of European ancestry, have reported associations between LTNP and HLA-B^∗^57, B^∗^27, B^∗^35, B^∗^58, and some HLA-C alleles (Goulder and Walker, 2012). HLA class I genotypes, however, vary substantially across populations, and the association between specific alleles and HIV-1 disease progression in African populations remains controversial, with previous studies mostly conducted in adult populations, showing country/region-specificity of results utilizing small sample sizes and candidate allele approaches (Serwanga et al., 2009; Matthews et al., 2011; Peterson et al., 2013; Sampathkumar et al., 2014; Shepherd et al., 2015). For example, HLA-B^∗^58:01 has been associated with slow progression in Uganda and South Africa (Serwanga et al., 2009; Payne et al., 2014), but this observation was not seen in Kenya and Botswana (Peterson et al., 2013; Payne et al., 2014); however, the initial Ugandan study was based on only 14 slow progressors, and the definition of LTNP was less stringent than comparable studies in Europeans (Serwanga et al., 2009). Notably, the HIV-1 epidemic in East Africa is predominantly due to HIV-1 subtypes A and D, while HIV-1 subtype C is predominant in southern Africa (Bbosa et al., 2019).

The now established protective effects of HLA-B^∗^57 in adults are also known to be population-specific - HLA-B^∗^57:01 is associated with control in Europeans while HLA-B^∗^57:03 is mostly strongly associated among Africans (Matthews et al., 2011). Most importantly, none of the canonical HIV-1 protective alleles (HLA-B^∗^57, B^∗^27, B^∗^35, B^∗^58) were replicated in a cohort of 123 HIV-1 clade C infected adolescents in Zimbabwe; instead, HIV-1 LTNP was attributed mainly to the HLA-C locus (HLA-C^∗^08:02 and -C^∗^08:04) (Shepherd et al., 2015). Adland and colleagues provided evidence of discordance in HLA-HIV associations between children and adults (Adland et al., 2015) in a South African cohort, among whom the putative protective effects of B^∗^57, HLA-B^∗^58:01, and HLA-B^∗^81:01 in 47 HIV-1 clade C infected mothers were non-existent in 84 children (Adland et al., 2015). HLA homozygosity has also been associated with rapid progression to AIDS, while heterozygosity has been associated with protection; however, HLA homozygosity is very low in Africa (Carrington et al., 1999; Cao et al., 2001; Peterson et al., 2013). Taken together, the current body of literature fails to provide a clear picture of the role of HLA alleles in pediatric HIV-1 LTNP, particularly among large African pediatric population groups (Thobakgale et al., 2009; Adland et al., 2015; Shepherd et al., 2015).

We have demonstrated uncaptured genetic variation in Botswana and Uganda (Retshabile et al., 2018) from exome sequencing data, which, alongside other studies of genetic variation across the continent (Sherman et al., 2019a; Choudhury et al., 2020), suggest a potential role for novel genetic variants in disease susceptibility and argue for the use of multi-ethnic African populations in such studies (Sherman et al., 2019b). We hypothesized that looking for genetic association between HLA and pediatric LTNP in multi-ethnic African populations could yield novel HLA variant associations, which might be relevant for epitope-based vaccine designs. To evaluate this, we inferred HLA class I alleles from exome sequencing data derived from a cohort of about ∼800 pediatric HIV-1 positive children from Uganda and Botswana, and determined evidence for an association between HLA class I variants and LTNP status.

## Materials and Methods

### Study Populations and Design

The Collaborative African Genomics Network (*CAfGEN*) is a collaboration between the Baylor International Pediatric AIDS Initiative (BIPAI) network sites in Uganda, Eswatini, and Botswana, Makerere University, University of Botswana, and Baylor College of Medicine. The details of the primary cohort’s demographic and clinical characteristics have been described (Retshabile et al., 2018; Mwesigwa et al., 2021; Kyobe et al., under review). This study was approved by the School of Biomedical Sciences Institutional Review Board (IRB), Uganda National Council for Science and Technology, University of Botswana IRB, Botswana Health Research and Development Committee, and the Baylor College of Medicine IRB.

We conducted a retrospective case-control study based on two extreme phenotypes of HIV-1 disease progression; LTNP and RP (Peloso et al., 2016). LTNPs were defined as children perinatally infected with HIV-1 and asymptomatic for more than 10 years with a CD4 count below 500 cells/ml or CD4 above 25%. The RPs were defined as an AIDS-defining illness, two or more consecutive CD4 below 15%, and ART initiation within 3 years of perinatal HIV-1 infection. Using electronic medical records (EMR), we identified 1,000 participants who met the criteria and were stratified equally in both groups. All participants provided written informed consent or assent according to age.

### Whole-Exome Sequencing (WES) and HLA Allelotyping

DNA exome sequencing for this cohort has been described in detail elsewhere (Retshabile et al., 2018). Overall, at least 96% of the bases targeted were covered at >20× depth (Retshabile et al., 2018). 822 participants had successful WES in three batches using the Illumina HiSeq 2000, HiSeq 2500, and NovaSeq. Four-digit HLA typing was inferred from WES data using HLAreporter, whose precision ranges from 96 to 100% depending on the locus and sequencing quality (Huang et al., 2015). Briefly, HLAreporter achieves accurate high-resolution typing of HLA alleles in four steps. First, WES FastQ files containing a mixture of short reads were mapped on a comprehensive reference panel from the IMGT/HLA database (v3.33) of HLA polymorphism. Secondly, the filtered and mapped reads (≥90 bp) were classified into specific HLA genes. Thirdly, the classified short reads were assembled *de novo* using a TASR assembler into contigs. The final two steps involve contig size score-based contig-HLA alignment using two candidate databases to identify only perfectly matched alleles (step 4) and assign G groups (step 5). Only contigs with sequencing depth above 10× were used during alignment; this is stringent and improves the accuracy of HLAreporter (Huang et al., 2015).

#### Validation

We conducted validation of HLAreporter using Lamda micro-SSP kits (according to manufacturers guidelines) on 20 randomly determined samples and observed 95% (19/20) concordance with four-digit high-resolution typing.

#### Statistical Analysis

Categorical demographic characteristics were summarized as counts and compared using χ^2^ tests, while continuous variables were summarized as median (interquartile range, IQR) and compared using the Wilcoxon rank–sum test. HLA allele frequencies were determined by expectation-maximization methods using PyHLA and compared using the prop.test in R (Fan and Song, 2017). The gametic phase was reconstructed using the ELB algorithm and pairwise linkage disequilibrium (LD) was measured using the Pearson’s squared correlation coefficient *r*^2^ (or Hendrik’s D′ for global LD between loci) in PyPop or Arlequin (Excoffier and Lischer, 2010) which is less sensitive to allele frequencies. The *p*-value threshold for significant LD (*p* < 3.18 × 10^––6^) was derived from Fisher’s exact test and corrected for multiple testing^1^ (Lancaster et al. 2003, 2007). Allelic associations were performed for alleles with frequencies >1% in the cohort using Fisher’s exact test. Odds ratios were calculated with Haldane’s correction of Woolf’s method (Yang et al., 2020), assuming an additive effects model. Our preliminary study (Kyobe et al., under review), we found some differences between children classified as LTNP by gender and geographical origin. We performed logistic regression and included country (as proxy for the genetic differentiation by PCA and Fst (see Results) as well as differences in HIV-1 subtypes between Uganda and Botswana) and gender as covariates to account for these differences. To establish the influence of homozygosity and heterozygosity of the significant alleles on disease progression, we performed zygosity analysis in PyPop (Svejgaard and Ryder, 1994). We constructed two-by-four tables and performed Svejgaard tests to establish the degree of inter-dependence between alleles associated with LTNP (Fan and Song, 2017).

For haplotype analyses, we used the Haplo.Stats package (v1.7.9) (Martin and Martin, 2021). Haplotype frequencies were estimated using the haplo.em function. We tested the association between haplotypes expressed at a frequency >1% and LTNP under an additive effect model using the haplo.score function. The haplo.glm and haplo.cc functions were used to calculate odds ratios using generalized linear regression models relative to the most frequent haplotype, controlling non-genetic factors (gender and country). We estimated the population attributable risk (PAR) due to the significant alleles and haplotypes using odds ratios from the logistic regression models (Bruzzi et al., 1985) according to Levin’s formula: P⁢A⁢R=P⁢(OR-1)P⁢(OR-1)+1⁢X⁢ 100, where p denotes the frequency of alleles or haplotypes among LTNPs and OR, is the odds ratios from the logistic regression models (Levin, 1953). False discovery rate (FDR) was used to adjust *P* values for the number of alleles tested (Pcorrected, *Pc* < 0.05). All statistical analysis was performed using Stata13 and R software v1.2.5033 (Gutierrez, 2010).

## Results

A total of 1000 participants, equally drawn from both countries, were recruited to participate in this stage of the study. As summarized in Table 1, we found that gender and country were similar at the geographic level, but there were significant differences in the number of LTNPs and RPs in the two countries (*p* < 0.001). Consistent with clinical observations, there were more female LTNPs than male. By definition, the median time to progression among LTNPs is significantly different from RPs, and we confirmed this in our cohort (157 months vs. 17 months, *p* < 0.001, Wilcoxon rank–sum test). Exome sequencing was successful for 394 LTNPs (207 Uganda, 187 Botswana) and 420 RPs (162 Uganda, 258 Botswana), and this final set of 814 individuals was utilized for downstream association analyses. Eight (∼1%; 8/822) samples were excluded from further analysis because no allele was typed at any locus. A total of 68 HLA-A, 88 HLA-B, and 63 HLA-C alleles were detected in our cohort.

**TABLE 1:** Baseline characteristics among LTNPs and RPs.

Variables	LTNPs	RPs	*P*-value
**Country**			
Botswana	187 (47.5)	258 (61.4)	**<0.001**
Uganda	207 (52.5)	162 (38.6)	
**Gender**			
Female	228 (57.9)	192 (45.7)	**<0.001**
Male	166 (42.1)	228 (54.3)	
Age (yr)	16.6 (13.7–19.4)	9.6 (6.6–12.1)	**<0.001**
Time to progression (mo)	157 (138–176.5)	17.0 (10.0–25.0)	**<0.001**
**HLA Class I**			
A	55	57	0.963
B	65	63	
C	47	48	

*Bold indicates variables that are different.*

### Population-Specific HLA Alleles and Haplotypes Are Enriched in HIV-Infected Children From Uganda and Botswana

**FIGURE 1:**
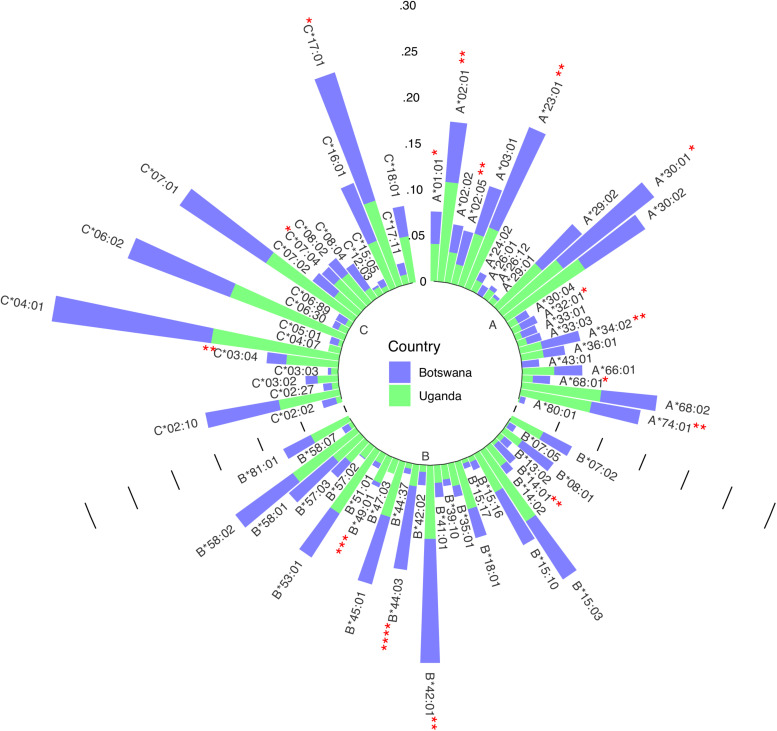
Circular bar plot of HLA class I distribution in Uganda and Botswana. Each bar represents a class I HLA allele. The heights represent the relative frequency of each allele per country and significantly different alleles are shown with asterisks.

**FIGURE 2:**
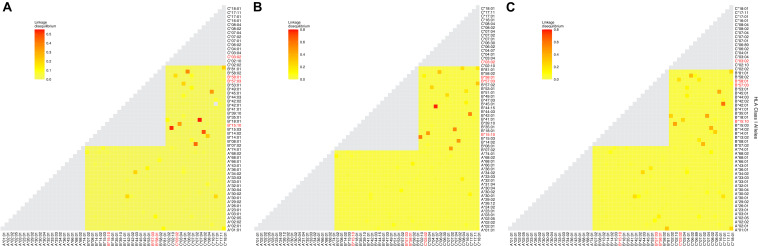
Patterns of linkage disequilibrium (LD) between the most frequent class I HLA alleles. LD in our cohort is expressed as the *R*^2^ values. LD plots were produced with **(A)** 56 alleles in the combined cohort, **(B)** 59 alleles in Uganda, and **(C)** 56 alleles in Botswana with a frequency ≥1%. The colors indicate increasing strength of LD from yellow to red. Alleles on the same HLA locus (no pairwise LD comparisons) are indicated in gray. Alleles associated with LTNP in the cohort are highlighted in red.

We have previously demonstrated low to moderate genetic differentiation between Uganda and Botswana populations using principal component analysis (PCA; using SNPrelate v1.10.2 in R see Supplementary Figure 1) and Fst based on Weir and Cockerham’s method using genome-wide data (Weir and Cockerham, 1984; Retshabile et al., 2018). The PCA plots (Supplementary Figure 1) and Fst of 0.0065 show some differentiation between the two populations at the global level of PC1 and 2, however, there is strong concordance at later PCs (PC1 vs. PC3) and the overall Fst differences are small. We started by exploring differences in HLA allele distributions (Figure 1) and pairwise LD between allele classes in the two countries (Figures 2A–C and Supplementary Tables 1–3). Six allelotypes - HLA-A^∗^30:01, −B^∗^42:01, −C^∗^04:01, −C^∗^06:02, −C^∗^17:01, and −C^∗^07:01 - all had allelotype frequencies above 10% in the full dataset (Figure 1). These alleles collectively accounted for 10, 11, and 52% of the total number of allelotypes at the HLA-A, −B, and −C loci, respectively (Figure 1 and Supplementary Table 4). Surprisingly, HLA-A^∗^30:01, B^∗^42:01, and C^∗^17:01 in Ugandans and C^∗^07:01 in Botswana were observed at between two and ten times higher sample frequencies, respectively, than previous studies of healthy Ugandan (*Z* test for proportions: *p* = 0.026, *p* = 0.002, and *p* = 0.006, respectively) and South African Black (*Z* test for proportions: *p* = 0.021) populations (Supplementary Table 5) (Cao et al., 2004; Tshabalala et al., 2018; Gonzalez-Galarza et al., 2020). This trend was similar when the data were analyzed by country (Supplementary Table 4). The frequencies of 16 alleles were significantly different between Uganda and Botswana (Figure 1 and Supplementary Table 6), with HLA-B^∗^44:03 being almost four times as common in Botswana as in Uganda (9.2 vs. 2.4%, *p* = 1.63 × 10^–4^). This allele is known to occur at low/uncommon frequencies in healthy populations worldwide, including Uganda and Zimbabwe (*p* = 0.005) (Cao et al., 2001, 2004; Kijak et al., 2009; Tshabalala et al., 2018; Gonzalez-Galarza et al., 2020), and has been independently associated with toxic epidermal necrolysis - a severe form of Stevens-Johnson syndrome (Ueta et al., 2014). HLA-B^∗^44:03-restricted epitopes have also been associated with asymptomatic chronic human cytomegalovirus (CMV) infection (Attaf et al., 2018).

We then examined global pairwise LD between the loci as a way of understanding the underlying HLA haplotype structure in our cohort. As expected, HLA-B and C loci were in strong LD (*D*′ = 0.867, Table 2), although quantitatively lower than reported in European ancestry cohorts (*D*′ = 0.928) (Cao et al., 2001; Tokić et al., 2020), this is consistent with LD in healthy African populations. In our combined cohort, the strongest LD was observed between HLA-B^∗^42:01 and C^∗^17:01 (*r*^2^ = 0.65, Figure 2A). While in Uganda and Botswana, HLA-B^∗^44:15 and C^∗^04:07 and HLA-B^∗^42:01 and C^∗^17:01 showed high LD values *r*^2^ = 0.78 and *r*^2^ = 0.61, respectively. Overall, both populations exhibited low LD patterns between class I HLA loci (Figures 2B,C).

**TABLE 2:** Global pairwise linkage disequilibrium.

HLA loci	Wn	D′	*P*-value
A:B	0.430	0.606	*p* < 0.0001*
A:C	0.372	0.552	*p* < 0.0001*
B:C	0.631	0.868	*p* < 0.0001*

Wn, Cramer’s V Statistic, D′ Hedrick’s statistic.

*p < 0.05 is indicative of overall significant LD.

**FIGURE 3:**
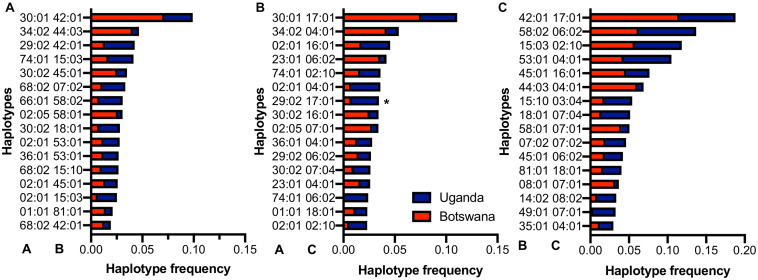
Distribution of the commonest HLA class I **(A)** A–B, **(B)** A–C and **(C)** B–C haplotypes in Uganda and Botswana. *-indicates alleles that are statistically different among both countries.

The number of bi-allelic haplotypes was not significantly different between Uganda and Botswana (Supplementary Table 7), suggesting a similar LD structure in the two countries. When we looked at the resulting pair-wise haplotypes, HLA-A^∗^30:01∼B^∗^42:01 (5.2%, *r*^2^ = 0.19), HLA-B^∗^42:01∼C^∗^17:01 (9.6%, *r*^2^ = 0.65) and A^∗^30:01∼C^∗^17:01 (5.6%, *r*^2^ = 0.19) were the most frequent across our two sample populations (Figure 3 and Supplementary Tables 8, 9). These four haplotypes have also been observed at high frequencies in healthy South Africans and Pumwani sex workers (Kloverpris et al., 2012; Sampathkumar et al., 2014), with HLA-B^∗^42:01-C^∗^17:01 being associated with faster seroconversion (rapid progression) in the Pumwani cohort (Sampathkumar et al., 2014). The frequency of these alleles and their concomitant haplotypes among healthy individuals of African ancestry (Ugandans, South Africans, Kenyans and Zimbabweans) is much lower than in healthy European and Hispanic groups (Cao et al., 2001; Kijak et al., 2009; Tshabalala et al., 2018), but they are enriched in HIV-1 infected populations generally (Sampathkumar et al., 2014); this may reflect enrichment of population- and disease-specific susceptibility factors (Supplementary Tables 6, 8). This pattern of shared haplotypes could be consistent with a codominant model of protection or susceptibility that might be relevant to migration patterns across the continent and the concomitant emergence of new pathogens.

### Established HLA-B Alleles and a Novel HLA-C Allele Show Association With LTNP in Ugandan and Batswana Children

**FIGURE 4:**
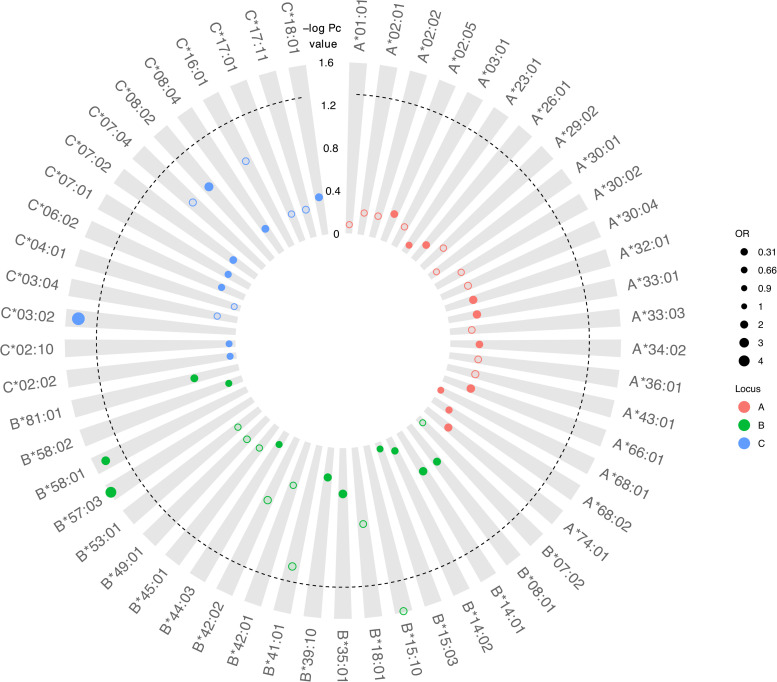
Circular bubble plot of the Class I HLA association with LTNP in the CafGEN cohort. Each bubble represents an HLA class I allele and the distance from the center is a measure of the negative log of the correct *P* (*Pc*) value. Protective alleles are represented with solid bubbles while the susceptible alleles are shown in blank bubbles. The size of the bubbles depicts the odds ratio from unity. The broken line represents the cut off of *Pc* value (<0.05).

**FIGURE 5:**
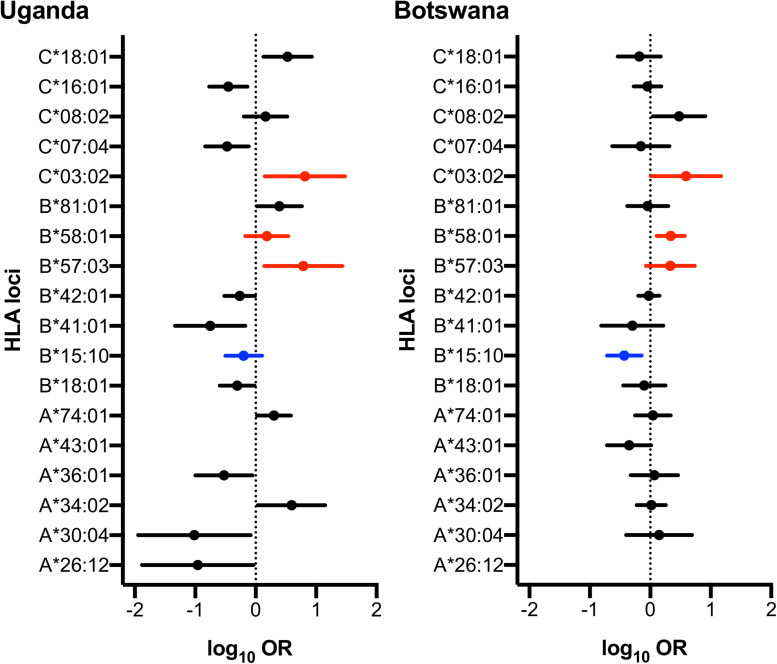
Forest plot of HLA alleles associated with LTNP in Uganda and Botswana. Only alleles found to be statistically associated with HIV progression in either country are shown. Alleles highlighted in red and blue have protective and detrimental effects, respectively, in both populations. Log Odds Ratios (95% CI) are obtained from logistic regression adjusting the effect of gender on HIV disease progression in each country.

Although there was no difference detected between LTNP (cases) and RP (controls) in the total number of alleles (*p* = 0.963, Table 1), among the 56 alleles with frequencies >1%, we identified three alleles with a significant positive association with LTNP status after adjusting for multiple testing and controlling for gender and country (*Pc* < 0.05, Figure 4 and Table 3) - HLA-B^∗^57:03 [3.9 vs. 1.2%, aOR 3.21 (95% CI 1.50–6.86), *Pc* = 0.026], HLA-B^∗^58:01 [6.6 vs. 3.9%, aOR 1.89 (95% CI 1.21–2.96), *Pc* = 0.033] and HLA-C^∗^03:02 [2.9 vs. 0.6%, aOR 4.74 (95% CI 1.74–12.85), *Pc* = 0.033] (Figure 4). Additionally, we found HLA-B^∗^15:10 [3.7 vs. 7.7%, OR 0.48 (95% CI 0.30–0.77), *Pc* = 0.0259] to be enriched among RPs (Figure 4 and Table 3) suggesting a predisposition toward rapid progression. These effects were generally consistent (in both magnitude and direction) across our two populations (Figure 5 and Supplementary Table 10). Most of these associated alleles were in weak LD (Figure 2A), suggesting that the associations observed are more likely to be independent. Given the moderate effect sizes observed in our cohort, we also investigated the population attributable risk due to the protective alleles; together, HLA-B^∗^57:03, B^∗^58:01, and C^∗^03:02 accounted for ∼16.5% (95% CI 3.5–40) of LTNP among children infected with HIV-1 in Uganda and Botswana.

**TABLE 3:** HLA alleles associated with LTNP in Uganda and Botswana.

HLA allele	Allele frequency in cohort				OR (95% CI)	*Pc* value^†^
	
	LTNPs	RPs	Total	*P*-value		
	(*n* = 393)	(*n* = 416)				
**Protective**						
B*57:03	0.038	0.012	0.025	0.0026	3.21 (1.50–6.86)	**0.025**
B*58:01	0.066	0.039	0.052	0.005	1.89 (1.21–2.96)	**0.033**
C*03:02	0.028	0.006	0.017	0.0022	4.74 (1.74–12.85)	**0.033**
C*08:02	0.042	0.019	0.030	0.0284	2.05 (1.07–3.90)	0.124
**Susceptible**						
B*15:10	0.037	0.077	0.057	0.0025	0.48 (0.30–0.77)	**0.025**
B*41:01	0.008	0.026	0.017	0.0139	0.31 (0.12–0.78)	0.069
C*07:04	0.021	0.036	0.029	0.0206	0.45 (0.23–0.88)	0.124
C*16:01	0.046	0.077	0.062	0.0333	0.62 (0.41–0.96)	0.124
B*18:01	0.031	0.051	0.041	0.0475	0.60 (0.36–0.99)	0.189

*Results of all observed HLA allele association per country are provided in Supplementary Table 10.*

*Bold indicates alleles that satisfy FDR correction *p* < 0.05.*

*^†^FDR adjusted *P*-value.*

**n*, number of participants.*

Next, we examined allelic zygosity, which influences susceptibility or protection from HIV-1 disease progression (Carrington et al., 1999). The overall homozygosity was consistent with previous reports, ranging from 5.3 to 8.6% at any one of the three loci; as expected, this was lower than the 13% reported in populations of European ancestry (Alter et al., 2017). Although there was no evidence for association with LTNP among participants who were homozygous for any of our putative protective alleles (Supplementary Table 11), we found that participants who were heterozygous at any of our four significantly associated alleles (HLA-B^∗^57:03, *Pc* = 0.0043; −B^∗^58:01, *Pc* = 0.0258; −C^∗^03:02, *Pc* = 5.7 × 10^–4^), and (B^∗^15:10 *Pc* = 0.0082) were significantly more likely to be LTNPs (Supplementary Table 11).

Next, we examined the association between common HLA haplotypes and LTNP status using multivariate regression, controlling again for the known effects of gender and country (Supplementary Table 12). We found two protective B∼C haplotypes and one susceptible A∼C haplotype with evidence of significant association. Globally, HLA haplotypes between B-C loci had the most statistically significant association with LTNP (global *p* = 0.006). Consistent with the single allele analyses, haplotypes B^∗^57:03-C^∗^07:01 [2.0 vs. 0.3%, OR 5.40 (95% CI 1.40–20.79), *p* = 0.025] and B^∗^58:01-C^∗^03:02 [2.2 vs. 0.5%, OR 4.88 (95% CI 1.50–15.86), *p* = 0.011] were over-represented and significantly associated with LTNP relative to the most frequent haplotype HLA-B^∗^42:01-C^∗^17:01 (Table 4 and Supplementary Table 12). Participants with the A^∗^29:02-C^∗^17:01 [1.0 vs. 2.9%, OR 0.26 (95% CI 0.08–0.79), *p* = 0.003] haplotype were 74% less likely to be LTNPs compared to individuals with the most frequent haplotype (HLA-A^∗^30:01-C^∗^17:01) (Table 4 and Supplementary Table 12). Taken together, the two observed protective haplotypes account for 9.8% (95% CI 1.0–35.2) of variation in disease progression attributed to LTNP in our cohort.

**TABLE 4:** Haplotype frequency and association with LTNP.

Haplotype*	Haplotype frequency in cohort			Haplotype effect^§^	
			
	LTNP	RP	*P*-value^†^	OR (95% CI)	*Pc* value
**Protective**					
B*57:03∼C*07:01	0.020	0.003	0.007	5.40 (1.40–20.79)	0.025
B*58:01∼C*03:02	0.022	0.005	0.004	4.88 (1.50–15.86)	0.011
		global *p* = 0.006			
**Susceptible**					
A*29:02∼C*17:01	0.010	0.029	0.008	0.26 (0.08–0.79)	0.003
		global *p* = 0.155			

*^†^The *p*-value is based on the individual haplotype association with LTNP compared to RP.*

*^§^ Odds ratios from generalized linear regression models comparing haplotype frequency with the most frequent haplotype and adjusted for country and gender.*

*In healthy population the haplotype frequencies of HLA−B^∗^57:03∼C^∗^07:01 are 1.4% in Uganda and 2.5% in Kenyan Nandi, HLA−B^∗^58:01∼C^∗^03:02 are 2% in Uganda and 4.2% in Kenyan Nandi and HLA−A^∗^29:02∼C^∗^17:01 are 2.5 and 0.352% in B^∗^42:01 containing haplotypes in Kenyan Nandi and black South Africans (Gonzalez-Galarza et al., 2020).*

### Protective Effects of HLA-C^∗^03:02 Appear to Be Additive and Independent of Linkage Disequilibrium

Our data suggested that the HLA-C^∗^03:02 allele is independently associated with a longer time to progression; however, the HLA-C^∗^03:02-HLA-B^∗^58:01 haplotype and the HLA-B^∗^58:01 allele were also associated with a longer time to progression in our cohort. This raised the question of the primary driver of our association. In our dataset HLA-C^∗^03:02 showed weak LD (*r*^2^ = 0.18) with HLA-B^∗^58:01, although this varied between LTNPs (*r*^2^ = 0.22) and RPs (*r*^2^ = 0.13), and between countries (Figure 2A and Supplementary Table 9). Therefore we sought to understand whether the effects of HLA-C^∗^03:02 were being mediated or augmented through interactions with B^∗^58:01. For this analysis, we utilized the interaction analysis proposed by Svejgaard in which the effect of the alleles under consideration are conditional upon co-inheritance (C03+/B58+) or not (C03+/B58− and C03−B58+) (Svejgaard and Ryder, 1994).

**FIGURE 6:**
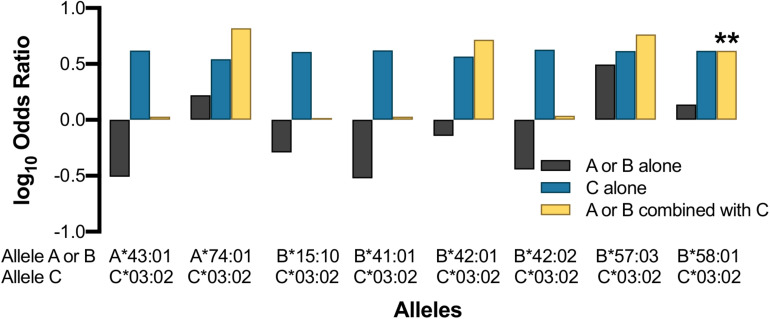
Effect of HLA-C*03:02 on other HLA class I alleles. Asterisk indicates allele combination that is statistically significant (*Pc* < 0.05) after correcting by a factor of 9 (Svejgaard and Ryder, 1994).

Surprisingly, the protective effect of HLA-C^∗^03:02 was not statistically significant in the absence of B^∗^58:01 (B58−/C03+; OR 4.15, *p* = 0.106; Figure 6), which was also observed for children expressing HLA-B^∗^58:01 without C^∗^03:02 (B58+/C03-OR 1.37, *Pc* = 0.311). We found that the protective effect of HLA-C^∗^03:02 remained even without concomitant co-inheritance of B^∗^58:01 (C03 + /B58−) although the statistical significance was reduced in accordance with the limited sample size (OR 4.15, *Pc* = 0.106); the reverse was also true (B58+/ C03−, OR 1.37, *Pc* = 0.311). Nevertheless, the co-expression of HLA-C^∗^03:02 and B^∗^58:01 (B58+/C03+) was associated with an effect size that was comparable with HLA-C on its own and was statistically significant [OR 4.15, *Pc* = 0.005 (*p* < 0.05/6 Svejgaard correction for established LD)]. This suggested that the effect of HLA-C^∗^03:02 was either dominant with respect to HLA-B^∗^58:01 or synergistic with it. To better understand the potential for synergistic effects, we looked for additive effects with other putatively associated alleles such as HLA-A^∗^74:01, which was marginally associated with LTNP in Uganda (Figures 4, 5 and Supplementary Table 10) and has been associated with viremic control (Matthews et al., 2011). Whereas HLA-A^∗^74:01 was marginally associated with LTNP in the absence of C^∗^03:02 (A74+/C03− aOR 1.66, *Pc* = 0.048), the combined protective effect of the haplotype was much stronger than either allele separately (OR 6.58, *Pc* = 0.0149). HLA-C^∗^03:02 and A^∗^74:01 are in very weak LD in our cohort (*r*^2^ = 0.02, Figure 2A), suggesting the effect of HLA-C^∗^03:02 was synergistic to the marginal effect of A^∗^74:01. Although our study was not fully powered statistically to detect epistatic differences, we consistently observed the emergence of a protective effect in all allelic combinations that included HLA-C^∗^03:02 with strongly protective (B^∗^57:03) as well as putatively detrimental (B^∗^42:01, A^∗^43:01, B^∗^15:10, B^∗^41:01) alleles (Figure 6), despite little LD between the alleles; this effect was not evident with overtly detrimental alleles. These data provide preliminary evidence that HLA-C^∗^03:02 may have a cooperative role when co-inherited with other protective alleles in African pediatric HIV-1 disease progression.

## Discussion

Using HLA typing derived from exome sequencing, we provide what to our knowledge is the largest survey of HLA allelotypes in pediatric HIV-1 infection in Africa. The sample sizes employed were more than sufficient to identify reported strong effects on disease progression. We confirmed previously known associations (HLA-B^∗^57:03 and B^∗^58:01) alongside a novel C^∗^03:02 association with LTNP. The effect sizes of the protective alleles and haplotypes ranged from 1.89 to 5.4, and, consistent with previous studies, these variants explain up to 16.5% of LTNP variation in our cohort. Our results expand upon the known and anticipated alleles and effect sizes in comparable HIV-1 infected pediatric populations in Africa (Shepherd et al., 2015).

The replication of previously reported HLA-HIV-1 associations (HLA-B^∗^57:03, B^∗^58:01, and B^∗^15:10) in African populations supports our methodological approach of using exome data for HLA typing in these cohorts. We observed slightly higher protective effects (OR 3.21) of HLA-B^∗^57:03 in our gender-mixed cohort than expected from the female sex workers cohort in Pumwani Kenya (OR 2.68) (Peterson et al., 2013). In Southern African adults, HLA-B^∗^57:03 and B^∗^58:01 have been associated with low set-point viral load and a higher CD4^+^ T-cell count during early infection - two phenotypes that positively correlate with LTNP status in adults (Tang et al., 2010; Chopera et al., 2011). Indeed, functional studies among adult LTNPs and elite controllers expressing B^∗^57:03 alleles have demonstrated that this allele presents epitopes that elicit superior CD8^+^ T-cell cytotoxicity activity (Migueles et al., 2015). These HLA-B associations are consistent with previous studies among adults infected with HIV-1 in Uganda, Zambia, Kenya, and South Africa (Serwanga et al., 2009; Leslie et al., 2010; Tang et al., 2010; Matthews et al., 2011; Peterson et al., 2013), as well as other Caucasian populations (Klepiela et al., 2004; Lazaryan et al., 2006; Chopera et al., 2011; Carlson et al., 2012; Gijsbers et al., 2013) and collectively, support the notion that heterozygous states of HLA protective alleles are advantageous, probably in both children and adults (Carrington et al., 1999; Arora et al., 2020), likely by providing alternative or diverse epitope presentation pathways especially in the emergence of HIV-1 escape mutants (Carlson et al., 2012; Gijsbers et al., 2013; Arora et al., 2020). This is the first report of HLA-B associations in pediatric HIV-1 infected populations from Africa (Adland et al., 2015; Shepherd et al., 2015).

The HLA-C^∗^03:02 allele was associated with a fourfold increase in LTNP status among carriers; a similar association has not been reported in LTNP studies in adults. HLA-C^∗^03:02 has been associated with a plethora of immune and immune-mediated phenomena, including methimazole-induced hepatotoxicity in patients treated for Graves disease (Li et al., 2019), lower BMI (Shen et al., 2018), and the development of eclampsia, allopurinol-induced SJS, and toxic epidermal necrolysis (Cristallo et al., 2011; van der Zwan et al., 2018). Other HLA-C alleles - C^∗^08:02 and C^∗^08:02 - have been reported as the main drivers of HIV-1 control in adolescents from Zimbabwe (*n* = 126) (Shepherd et al., 2015). In our cohort HLA-C^∗^08:02 did not reach statistical significance after correction for multiple testing (Table 3). HLA-C^∗^03:02 and C^∗^08:02/04 molecules differ at positions 35 (R35Q), 114 (D114N), 116 (S116F), and 163 (L165T), located in pockets B, C, D, and E, respectively of the protein binding groove, suggesting that these alleles may have differing peptide binding affinities for different HIV-1 epitopes (Kloverpris et al., 2012). Higher extracellular surface expression of HLA-C molecules has been proposed and demonstrated as the most likely mechanism in HLA-C-mediated delayed HIV-1 disease progression (Thomas et al., 2009; Apps et al., 2013), however, the mean expression of HLA-C^∗^03:02 is lower than C^∗^08:02 (Apps et al., 2013) on CD3^+^ cells; therefore there may be additional mechanisms through which HLA-C^∗^03:02 can control HIV-1 in African children, such as microRNA regulation of HLA-C expression and KIR recognition (Kulkarni et al., 2011; Li et al., 2018; Vargas et al., 2020).

HLA-C^∗^03:02 was found in weak LD with B^∗^58:01 (*r*^2^ = 0.18), and both were associated with long-term control of HIV. Interestingly, the haplotypic effect of HLA-C^∗^03:02 in combination with B^∗^58:01 is similar to the individual allelic effect of C^∗^03:02 (OR 4.88 vs. 4.78), and the allelic effect of carrying C^∗^03:02 is twice that seen among carriers of B^∗^58:01 (OR 4.68 vs. 1.72). However, our analysis of carriers of C^∗^03:02 without B^∗^58:01 showed that the associations were lost despite the strong association with protection observed when the allele was assessed agnostic to other alleles or as part of a haplotype. HLA-C^∗^03:02 also showed an apparent additive effect when combined with other putatively protective alleles such as HLA-A^∗^74:01, and this apparent synergistic effect of C^∗^03:02 was consistently observed in combinations with detrimental alleles (Figure 6). These observations, coupled with the overall heterozygous advantage of the HLA-C^∗^03:02 allele (*p* = 5.7 × 10^–4^), lead us to postulate that HLA-C^∗^03:02 may play a synergistic role in LTNP in our population groups.

We only observed a single susceptible allele and haplotype (enriched among rapid progressors) in our cohort - we confirm a previous independent association between HLA-B^∗^15:10 and rapid progression with a 55% reduction in the odds of LTNP being observed in our cohort. This is congruent with prior data from Kenya (OR 0.45 vs. 0.49) (Peterson et al., 2013), which may partially reflect the predominant HIV-1A and D clades seen in both Uganda and Kenya (Sampathkumar et al., 2014). This association was also seen in Botswana, where the predominant clade is HIV-1C (Serwanga et al., 2009; Payne et al., 2014).; some HLA alleles are known to bind and present epitopes from multiple clades [allelic promiscuity (Chappell et al., 2015)], which could explain the broad applicability of this observation, as such alleles could have poor specificity and so potentiate viral replication (Honeyborne et al., 2006). HLA-B^∗^15:10 was found to be expressed at lower levels in cells deficient of transporter associated with antigen processing 1 (TAP1) and TAP2, implying that B^∗^15:10 might utilize TAP-dependent pathways for loading epitopes (Geng et al., 2018). TAP-dependent pathways are frequent targets of viral immune evasion (Verweij et al., 2015). Haplotype A^∗^29:01-C^∗^17:01 was also associated with rapid progression; consistent with Sampathkumar et al. who found that a C^∗^17:01 containing haplotype was associated with faster HIV-1 seroconversion among sex-workers in Kenya (Sampathkumar et al., 2014).

Some limitations of our study merit mention. Given the long-term follow-up in our cohort, the study is limited by survival bias, therefore, the associations observed could be due to enrichment of protective HLA variants. HLA genotypes were inferred from exome sequence data, as opposed to other sequence-based or sequence-specific primer typing techniques. Despite the enrichment for exonic regions in the genome during exome sequencing, the high polymorphism in the HLA region makes such data less reliable, potentially leading to misclassification. Current evidence, however, suggests that this may not be as significant a concern as initially feared (Duke et al., 2016; Sverchkova et al., 2019; Tokić et al., 2020), and we observed a high degree of concordance between inferred and allelotyped alleles in our validation experiments. While HWE checks are useful for validating genotypes/genotyping in disease-free controls, we did not have access to suitably matched healthy cohorts and deviations from HWE are not always sufficiently sensitive to case ascertainment status, particularly with dense datasets. Instead we performed a limited validation of the results using an orthogonal method of allelotyping, which showed almost perfect concordance with our inferred allelotypes. Thus, we do not believe the allelotyping to be generating false positive results. Given the modest sample sizes employed, we were underpowered to stratify the haplotype analyses by country; independent replication of our findings in other larger pediatric populations is thus highly desired. Unfortunately, WHO changes on ART initiation (test and treat strategy) (Kretzschmar et al., 2013) mean that such studies will necessarily need to rely upon large retrospective datasets, which are relatively scarce within this age group.

The primary objective of our study was to evaluate enrichment of class I HLA loci on HIV disease progression (rather than HIV acquisition); necessarily, this required all participants to have the exposure of being HIV positive. A better context for our results would be comparison of allele frequencies among unaffected (normal) individuals from our population groups; however, there are no unaffected pediatric cohorts from our cohort countries, and comparisons across generations (adults vs. children), particularly in the context of HIV where there is likely to be ascertainment differences between sites, are made with trepidation. None-the-less, we provide population frequencies of the highlighted alleles from healthy available populations for completeness (Supplementary Table 5). Unlike Uganda, there is no comparable HLA frequency data from Botswana in the Allele Frequency Database (AFND) (Gonzalez-Galarza et al., 2020). We therefore chose to use South African Blacks or Zimbabwe Shona from the AFND and adult data from Uganda and Kenya (Luo and Nandi) as proxy populations for our Botswana and Uganda cohorts, respectively. In Botswana, this is undoubtedly imperfect, although the black South African populations are believed to be closely related to the Tswana people in Botswana, with some evidence of linguistic links across countries (Table 4 and Supplementary Table 5).

Overall, the protective alleles and haplotypes reported here account for <20% of LTNP in our cohort, leaving more than two-thirds of LTNP unexplained. Therefore, we believe that as yet unrecognized host and other factors could play key roles in determining HIV-1 progression in African children. For instance, we have recently documented an abundance of Anelloviridae viral species among LTNPs in our cohort (Mwesigwa et al., 2021). Anelloviridae are thought to impact NK cell activity through NF-kB, and, since HLA is known to interact with killer immunoglobulin-like receptors (KIR) on natural killer (NK) cells, the synergy between these two mechanisms could provide an alternative pathway for clearance of virally infected cells via NK cell cytotoxicity (Paximadis et al., 2011). Exploring these and other factors in our population cohorts may yield additional factors that mediate pediatric LTNP status in Africa and provide a potential path to new vaccines and therapeutics.

## Conclusion

We provide evidence for the benefit of geographically independent multi-ethnic African populations to unravel novel HLA and HIV-1 disease associations. We confirm known and identify novel HLA associations with LTNP in two African pediatric populations. Consistent with emerging evidence, we demonstrate the role of HLA-C in the control of HIV-1 infection in children. Our results bolster a growing body of literature in support of an important role for HLA-C alleles in the control of HIV-1 among children and adolescents.

## Data Availability Statement

The datasets presented in this study can be found in online repositories. The names of the repository/repositories and accession number(s) can be found below: https://h3africa.org/wp-content/uploads/2018/05/App-D-H3Africa-Data-and-Biospecimen-Access-Committee-Guidelines-final-10-July-2017.pdf, NA.x.

## Ethics Statement

The studies involving human participants were reviewed and approved by Makerere University School of Biomedical Sciences IRB, Uganda National Council for Science and Technology (UNCST), University of Botswana IRB, Botswana Health Research and Development Committee (HRDC), and Baylor College of Medicine IRB. Written informed consent to participate in this study was provided by the participants’ legal guardian/next of kin.

## Author Contributions

SK, MW, MJ, GK, AK, BN, MM, GA, IK, SWM, MT-J, CB, GM, and NH: conceptualization, writing – review and editing, funding acquisition, investigation, and project administration. SK, SM, ErK, MW, FK, AM, BN, JF, GR, BM, LW, and KM: data curation. SK, SM, and NH: formal analysis. JK-L: funding acquisition. EW and JK-L: investigation. SK, GK, GR, BN, MM, CB, GM, and NH: methodology. EW: project administration. GM, NH, MJ, AK, MM, and JK-L: supervision. SK and NH: validation and writing – original draft. All authors contributed to the article and approved the submitted version.

## Conflict of Interest

The authors declare that the research was conducted in the absence of any commercial or financial relationships that could be construed as a potential conflict of interest.

## Publisher’s Note

All claims expressed in this article are solely those of the authors and do not necessarily represent those of their affiliated organizations, or those of the publisher, the editors and the reviewers. Any product that may be evaluated in this article, or claim that may be made by its manufacturer, is not guaranteed or endorsed by the publisher.

## Footnotes

1
https://www.hiv.lanl.gov/content/index
