# Supplementary figures and images for: Exome Sequencing Reveals a Putative Role for HLA-C*03:02 in Control of HIV-1 in African Pediatric Populations
Source: Front Genet. 2021 Aug 26;12:720213. doi: 10.3389/fgene.2021.720213 (PMC8428176; doi:10.3389/fgene.2021.720213)

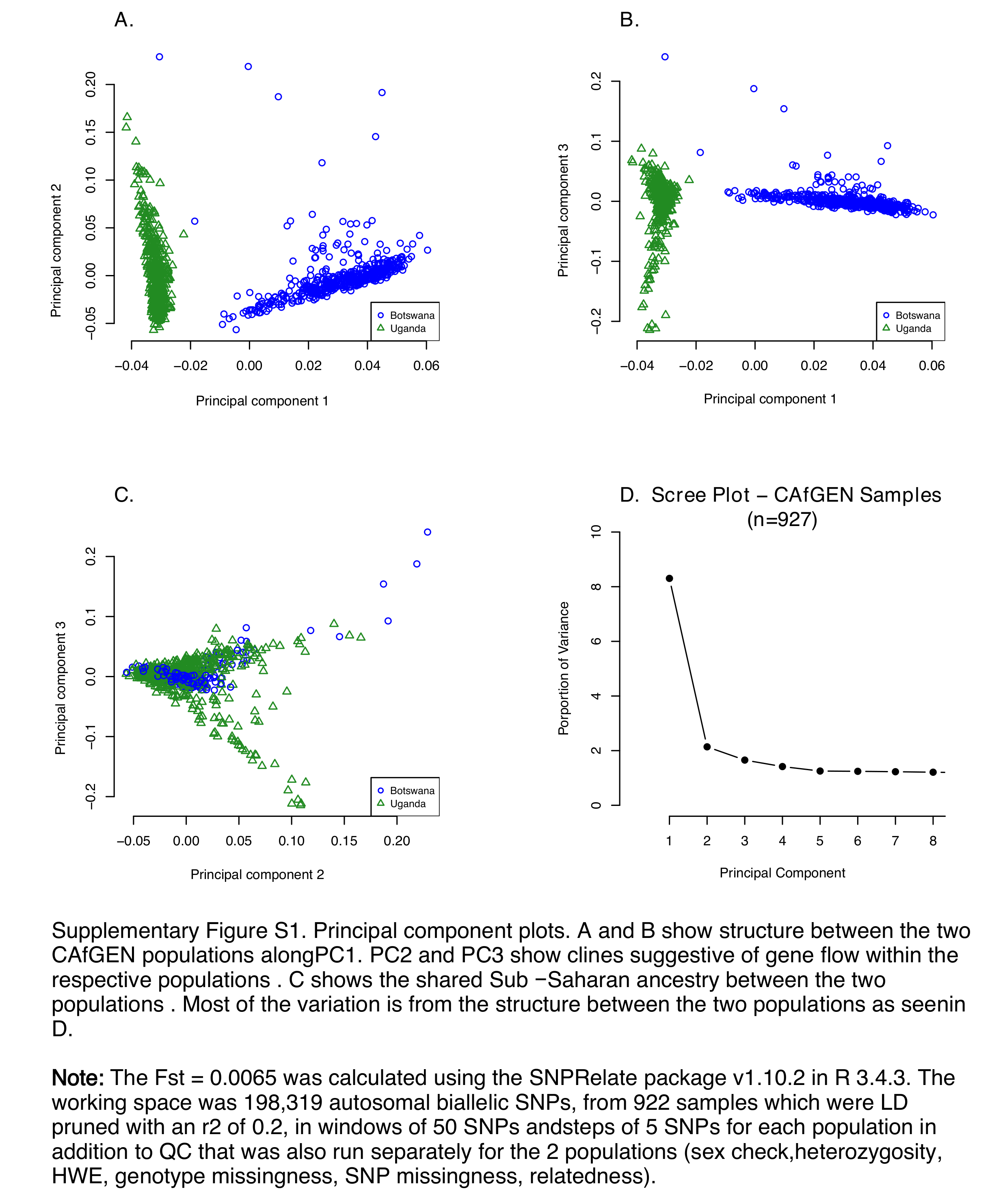

Supplement: Supplementary file 1 [file Image_1.TIF]
